# Complementary Use of Cultivation and High-Throughput Amplicon Sequencing Reveals High Biodiversity Within Raw Milk Microbiota

**DOI:** 10.3389/fmicb.2020.01557

**Published:** 2020-07-09

**Authors:** Franziska Breitenwieser, Etienne V. Doll, Thomas Clavel, Siegfried Scherer, Mareike Wenning

**Affiliations:** ^1^Milchprüfring Baden-Württemberg e.V., Kirchheim unter Teck, Germany; ^2^Chair for Microbial Ecology, Weihenstephan School of Life Sciences, Technische Universität München, Freising, Germany; ^3^Functional Microbiome Research Group, Institute of Medical Microbiology, RWTH University Hospital, Aachen, Germany; ^4^ZIEL – Institute for Food and Health, Technische Universität München, Freising, Germany; ^5^Bavarian Health and Food Safety Authority, Oberschleißheim, Germany

**Keywords:** raw milk, biodiversity, microbiota, 16S rRNA gene amplicon sequencing, cultivation

## Abstract

Raw milk microbiota are complex communities with a significant impact on the hygienic, sensory and technological quality of milk products. However, there is a lack of knowledge on factors determining their composition. In the present study, four bulk tank milk samples of two farms at two different time points were analyzed in detail for their microbiota using cultivation and 16S rRNA amplicon sequencing. Diversity in samples from the first time point was assessed via cultivation of 500 aerobic mesophilic bacterial isolates in each sample. A high biodiversity of 70 and 110 species per sample was determined, of which 25–28% corresponded to yet unknown taxa. The isolates were dominated by Gram-positive members of the genera *Staphylococcus, Corynebacterium, Streptococcus*, or *Janibacter*, whilst *Chryseobacterium* and *Acinetobacter* were most abundant among the Gram-negative taxa. At the second time point, samples of the same farms were analyzed via both cultivation (1,500 individual colonies each) and high-throughput 16S rRNA gene amplicon sequencing. The latter revealed a threefold higher biodiversity at the genus level, as anaerobic or fastidious species were also detected. However, cultivation identified genera not captured by sequencing, indicating that both approaches are complementary. Using amplicon sequencing, the relative abundance of a few genera was distorted, which seems to be an artifact of sample preparation. Therefore, attention needs to be paid to the library preparation procedure with special emphasis on cell lysis and PCR.

## Introduction

The indigenous microbiota of raw milk has re-gained attention during the last years for economic and safety reasons. Consumers increasingly prefer unpasteurized products such as raw milk cheese or raw milk bought in retail sale or directly at the farm. This increases the risk of zoonotic diseases due to the presence of pathogenic bacteria like *Listeria monocytogenes*, *Salmonella* spp.,*Campylobacter* spp. or Shiga toxin-producing *Escherichia coli* (Giacometti et al., 2013; Gould et al., 2014; Christidis et al., 2016; Artursson et al., 2018; Willis et al., 2018). In the United States, unpasteurized milk and cheese, which are consumed by a minority of the population only, caused 96% of outbreak-related illnesses because of contaminated dairy products between 2009 and 2014 (Costard et al., 2017). Health risks are largely diminished by heat treatment of milk, but spoilage as a result of heat-resistant microorganisms or enzymes can still occur. Spore-formers are responsible for the major part of spoilage in pasteurized milk (Huck et al., 2008; Schmidt et al., 2012; Doll et al., 2017). Even UHT milk, although microbiologically stable, can be indirectly affected by the raw milk microbiota. Heat-resistant peptidases primarily produced by Gram-negative bacteria such as *Pseudomonas* or *Serratia* (Marchand et al., 2009; von Neubeck et al., 2015; Machado et al., 2017) impact the sensory and textural properties of UHT milk during elongated shelf-life and even very low residual activities may lead to bitterness and age gelation (Stoeckel et al., 2016). Thus, different product types are affected by different parts of the microbiota that are not eliminated during processing. However, besides all negative implications, the raw milk microbiota is valuable and particularly appreciated, for instance for obtaining individual and flavor-rich cheeses (Callon et al., 2005; Montel et al., 2014).

To improve consumer safety, maintain tasteful product quality, achieve a longer shelf-life, and optimize the use of beneficial bacteria in manufacturing of raw milk dairy products, the complex inter-play of farming practices, animal physiology and composition of raw milk microbiota needs to be understood. This will enable the development of housing and milking strategies effective in reducing undesired bacteria whilst maintaining a stable and beneficial microbiota. Different factors influencing the milk microbial communities have been examined over the last decades (Desmasures and Gueguen, 1997; Christiansson et al., 1999; Vacheyrou et al., 2011; Mallet et al., 2012; Verdier-Metz et al., 2012; Miller et al., 2015), but these studies relied all on laborious culture-dependent methodologies and, therefore, were limited in size, sensitivity, and focus. The emergence of high-throughput sequencing techniques has paved the way for much more comprehensive investigations, which led to a multitude of analyses published during the last few years (Quigley et al., 2013; Oikonomou et al., 2014; Ganda et al., 2016; Kable et al., 2016; Bonsaglia et al., 2017; Doyle et al., 2017a, b; Rodrigues et al., 2017; Fretin et al., 2018; Li et al., 2018; Lima et al., 2018). However, as demonstrated for other microbial communities such as those in the gut of mammals, sequencing approaches do have their own limitations and cultivation is still a valuable tool (Browne et al., 2016; Clavel et al., 2016; Clooney et al., 2016). To be able to link the outcomes of new investigations with former results, it is necessary to use culture- and sequence-based approaches concomitantly, thereby revealing outcome similarities and divergences between methodologies. In addition, the power and limits of culture-based approaches to detect microbial biodiversity in raw milk was never studied in detail.

The main objective of this study was a deep analysis of raw milk biodiversity by using a combination of cultivation, focusing on aerobic taxa including food-borne pathogens and potential spoilage organisms, and high-throughput 16S rRNA gene amplicon analysis.

## Materials and Methods

### Collection of Milk Samples

Bulk tank milk of two farms (A and B) in Southern Germany with a herd size of about 45 animals was collected, each on two occasions. The first time, both farms were sampled simultaneously in January (samples 1 and 2). Samples were automatically taken from the bulk tank containing four to six successive milkings using the sampling device of the milk collecting vehicle, transported to the laboratory under refrigerated conditions (maximum 6°C) within 24 h and processed within 1 h after arrival. The second time, each farm was sampled independently, farm A (sample 3) 26 months and farm B (sample 4) 18 months after the first sampling. Milk was collected directly from the bulk tank containing one and two milkings, respectively. The coupling pipe was disinfected with 70% ethanol and rinsed with milk from the tank before samples were collected. Milk was transported under refrigerated conditions and analysis started 3–4 h after sample collection. Part of the milk was used immediately for the culture-dependent analysis, whereas 150 mL were frozen and stored at −80°C for molecular work.

### Bacterial Counts

Total aerobic counts (TAC) of the mesophilic raw milk microbiota were determined using the spread-plate method. Decimal dilutions were plated on tryptic soy agar (TSA, Oxoid) with 10 plates per dilution and incubated at 30°C for 5 days.

### Culture-Dependent Analysis of Raw Milk Microbiota

In the first approach (sample 1 and 2), 500 colonies were randomly isolated from agar plates used for TAC determination. All colonies were selected from the identical dilution step with plates showing well-separated colonies. Starting with the first petri dish, all colonies present on this dish were picked and sub-cultured on TSA. The procedure was repeated until a total of 500 colonies was obtained. Isolates were identified using FTIR spectroscopy and gene sequencing for representative isolates. In the second approach (samples 3 and 4), the same isolation procedure was followed with the exception that larger petri dishes (Ø = 15 cm) were used to apply larger sample volumes and obtain higher numbers of isolates: 1,500 colonies were selected per sample and all were identified by gene sequencing.

#### Identification of Isolates by FTIR Spectroscopy

Sample preparation and recording of spectra was performed as described previously (Wenning et al., 2014; von Neubeck et al., 2015). Identification of spectra was conducted using in-house reference databases containing approximately 8,000 spectra of 1,000 species using the parameters described by Kümmerle et al. (1998) and Oberreuter et al. (2002). To reduce redundancy, FTIR spectra of all isolates were compared for each sample by hierarchical cluster analysis (HCA) as described previously (von Neubeck et al., 2015). Representative isolates were selected and subsequently identified by gene sequencing.

#### Sanger Sequencing

For identification of most bacterial isolates, the 16S rRNA gene was used. The *rpoB* gene (Mellmann et al., 2006) was used for *Staphylococcus* spp. and the 26S rRNA gene for yeasts (Kurtzman and Robnett, 2003). Cell lysis, PCR, and sequencing were performed as described previously (von Neubeck et al., 2015). *rpoB* and 26S RNA gene sequences were identified using the NCBI database, 16S rRNA gene sequences using EzBiocloud (Yoon et al., 2017). Thresholds for species and genus assignment of 16S rRNA gene sequences were 98.65 (Kim et al., 2014) and 95% sequence similarity, respectively; 95% similarity was used for species assignment of *rpoB* sequences.

### High-Throughput 16S rRNA Gene Amplicon Analysis of Raw Milk Microbiota

Milk samples 3 (farm A) and 4 (farm B) subjected to culture-based analysis of 1,500 colonies were also used for amplicon sequencing.

#### Concentration of Microbial Cells in Raw Milk

As microbial loads in fresh raw milk are comparatively low and did not exceed log 5 cfu/mL in samples 3 and 4, bacterial cells were concentrated prior to DNA extraction. A volume of 150 mL was centrifuged at 8,000 × *g* for 20 min at 4°C to pellet bacterial cells. The supernatant consisting of a fat layer and skim milk was carefully removed, except for 10 mL skim milk, which were left to re-suspend the pellet. The suspension was transferred to a 50 mL tube and additionally centrifuged at 5,000 × *g* for 10 min at 4°C. Again, the fat layer was carefully removed.

#### Removal of Sedimented Casein

Sedimented casein was removed according to Murphy et al. (2002). The cell suspension was divided into 10 aliquots of 1 mL and 300 μL 0.5 M EDTA (pH 8.0) as well as 200 μL TE buffer (pH 7.6) were added to each aliquot. Within approximately 1 min, the casein micelles disintegrated due to chelating of calcium ions indicated by clarification of the solution, which was centrifuged at 16,000 × *g* for 1 min at room temperature. The supernatant was discarded and the pellet re-suspended in 100 μL Ringer’s solution. All ten subsamples were then pooled and centrifuged again. The supernatant was discarded and the pellet re-suspended in 350 μl quarter strength Ringer’s solution.

#### DNA Extraction and Enrichment of Bacterial DNA

The PathoProof DNA Extraction Kit (Thermo Fisher Scientific), including enzymatic lysis of microbial cells, was applied according to the manufacturer’s instructions to extract DNA, producing a final volume of 100 μL DNA extract per sample. As bulk tank milk contains high amounts of eukaryotic DNA originating from somatic cells of the cow, enrichment of bacterial DNA using the Looxster Enrichment Kit (Analytik Jena, Germany) was performed according to the manufacturer’s instructions resulting in 30 μL of DNA for each sample.

#### Determination of Bacterial DNA Concentrations

The concentration of total DNA was determined using the Qubit^®^ 2.0 fluorometer (Life Technologies Co.) using the dsDNA HS Assay Kit. In a further step, the concentration of bacterial DNA in both extracts was determined using quantitative real-time PCR. PCR mixtures contained 4 μL Phusion^®^ Buffer HF (Thermo Fisher Scientific Inc.), 1 μL dNTPs (20 nM), 2 μL each of primer 515F (5′-GTGCCAGCMGCGCGGTAA) and 806R (5′-GGACTACHVGGGTWTCTAAT) (10 pmol/μL), 0.1 μL Phusion High-Fidelity DNA Polymerase (Thermo Fisher Scientific Inc.), 1 μL SYBR-Green (diluted 1:50,000), and 2 μL of DNA extract (diluted 1:10) in a final volume of 20 μL. After initial denaturation at 98°C for 30 s 40 cycles of 5 s denaturation at 98°C, 10 s primer annealing at 52.5°C, and 10 s elongation at 72°C were run. A DNA standard composed of somatic DNA extracted from bulk tank milk and different fractions (10, 1, and 0.1%) of a bacterial DNA extract of a *Pseudomonas* and *Enterococcus* raw milk isolate was used for quantification of bacterial DNA.

#### Library Preparation for Amplicon Sequencing

DNA quantification resulted in a total DNA concentration of 15 ng/μL for sample 3 of farm A and 10 ng/μL for sample 4 of farm B. Sample 3 contained 4.8% (0.7 ng/μL) bacterial DNA, sample 4 2.5% (0.25 ng/μL). DNA extracts were then standardized to contain 0.2 ng/μL bacterial DNA. Library preparation followed a two-step protocol (Berry et al., 2011) based on amplification of the V3–V4 region of the 16S rRNA gene using primers 341F (5′-CCTACGGGNGGCWGCAG) and 785R (5′-GGATTAGATACCCBDGTAGTC) (Klindworth et al., 2013). Due to the low concentrations of template microbial DNA, the PCR protocol was adjusted and the number of replicates as well as the number of cycles augmented. For the first PCR step, eight parallel PCRs (four barcodes in duplicate) were run for each sample, each reaction using 3.5 μL DNA extract containing 0.7 ng bacterial DNA. After initial denaturation at 98°C for 30 s, 30 cycles of 5 s denaturation at 98°C, 10 s primer annealing at 55°C, and 10 s elongation at 72°C were run. The second PCR step added barcodes (dual combinatorial indexing) and Illumina adaptors to the amplified fragments and was done using the same protocol but 2 μl of PCR product of step 1 as template and was run only for 10 additional cycles. A negative control (PCR blank) was included using PCR-grade water as template.

#### Purification of Amplified DNA and Sequencing

Duplicate PCRs for any given samples (i.e., having the same barcodes) were transferred into a 1.5 mL tube and purified using Agencourt AMPure XP Beads (Beckman Coulter Inc.). DNA concentrations were determined via Qubit^TM^ 2.0 fluorometer and adjusted to 2 nM. All libraries were pooled and sequencing was performed in paired-end mode (2 × 275 cycles) using a MiSeq platform (Illumina).

#### Analysis of Sequencing Data

Raw reads were processed using the IMNGS pipeline (Lagkouvardos et al., 2016) based on UPARSE (Edgar, 2013). After demultiplexing, forward and reverse reads were merged and trimmed by five nucleotides on each end. Chimera filtering was done using UCHIME (Edgar et al., 2011). Operational Taxonomic Units (OTU) clustering (97% identity) was performed by USEARCH 8.0 (Edgar, 2010) and OTUs occurring at a relative abundance <0.05% in all samples were discarded. Taxonomical identification was assigned using the RDP classifier (Wang et al., 2007) and diversity analyses were done in Rhea (Lagkouvardos et al., 2017). Relative abundances of OTUs were normalized to account for differences in sequence depth and α-diversity was assessed on the basis of species richness as well as Shannon and Simpson diversity indices. As automated identification may be imprecise and often terminates at higher ranks, 487 OTU sequences not assigned to a genus were manually identified at the genus level (95% similarity) using EzBiocloud (Yoon et al., 2017). OTUs for manual identification were selected according to the following criteria: all OTUs that were identified only to the phylum level or above, all OTUs belonging to families detected by culturing, and the 100 most abundant OTUs in each sample. ß-diversity was computed for both the culture-dependent and -independent results based on generalized UniFrac distances (Chen et al., 2012) and visualized in a phylogram based on the Ward’s minimum variance method (Murtagh and Legendre, 2014).

## Results

Bulk tank milk of two farms in Southern Germany was analyzed in detail for microbial biodiversity. In a first culture-dependent approach, 500 colonies per sample were isolated and identified. In a second approach, milk of the same farms was analyzed based to 1,500 colonies per sample combined with high-throughput sequencing.

### Culture-Dependent Analysis of 500 Isolates

**FIGURE 1:**
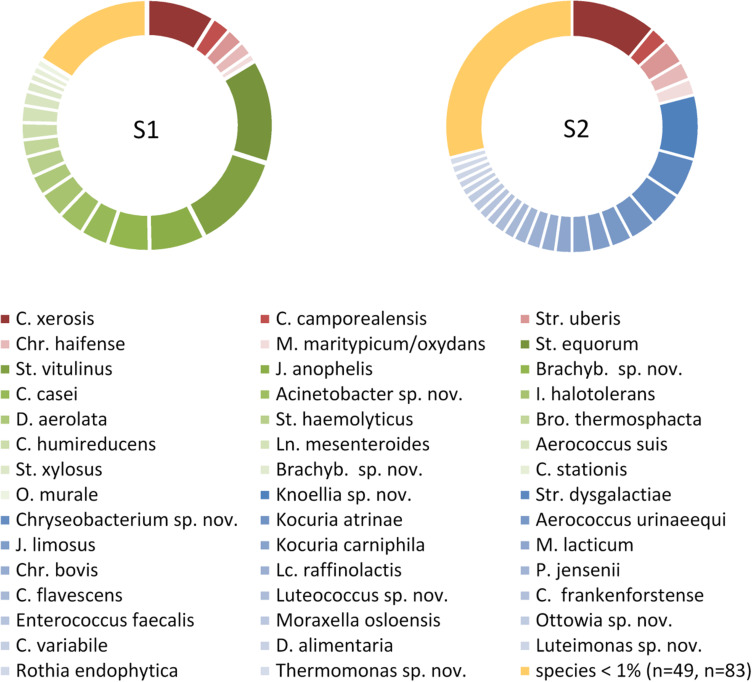
Composition of the microbiota in two bulk tank milk samples based on each 500 isolates. Only species with a relative abundance >1% of the total isolates are listed. Species occurring in only one sample are indicated in green or blue, species detected in both samples are given in red. S1, sample 1; S2, sample 2; Brachyb., *Brachybacterium*; Bro., Brochotrix; C., *Corynebacterium*; Chr., *Chryseobacterium*; D., *Dietzia*; I., *Isoptericola*; J., *Janibacter*; Ln., *Leuconostoc*; Lc., *Lactococcus*; M., *Microbacterium*; O., *Ornithinimicrobium*; P., *Propionibacterium*; St., *Staphylococcus*; Str., *Streptococcus*; sp. nov., hitherto undescribed species.

The sample from farm A (sample 1) contained six, the one of farm B (sample 2) four successive milkings and both had a TAC of 4.7 log cfu/mL. We identified 495 (sample 1) and 472 (sample 2) isolates to the species level revealing a high biodiversity. Sample 2 contained 110 species assigned to 57 genera, whereas sample 1 was a little less diverse with 71 species belonging to 36 genera (Figure 1 and Table 1). Most of the diversity was represented by only a minor fraction of isolates, as the majority of species occurred with a relative abundance <1%. In sample 1, 49 species (69%) were represented by 16% of isolates and in sample 2, 83 species (75%) accounted for 29% of isolates (Figure 1 and Supplementary Tables S1, S2). Both microbiota were dominated by Gram-positive taxa (Figure 1 and Supplementary Tables S1, S2). Isolates from Gram-negative species accounted for less than 10% in sample 1 and little more than 20% in sample two. Many species in sample 1 belonged to *Staphylococcus* and *Corynebacterium* (8 and 10, respectively, Supplementary Table S1), many of which were among the most abundant species. In sample 2, 14 species belonged to *Corynebacterium*, but the genus with the second most species was *Chryseobacterium* (Supplementary Table S2). The fact that 40% of all species were detected with only a single isolate (relative abundance 0.2%) indicates that the biodiversity present was insufficiently covered and a substantial fraction of species remained undiscovered.

**TABLE 1:** Microbial biodiversity of samples 1–4 based on the cultivation of isolates on TSA.

	Farm A		Farm B	
Sample	1	3	2	4
No. milkings	6	1	4	2
TAC (log cfu/mL)	4.65	4.32	4.70	4.40
No. bacterial isolates	498	1,475	501	1,102
No. genera	36	47	57	60
No. hitherto unknown genera	2	2	5	3
No. genera with relative abundance ≥1% (% of isolates)	16 (92.4)	14 (91.2)	24 (86.2)	21 (90.7)
No. species	71	112	110	125
No. hitherto unknown species	21	23	28	24
No. species with relative abundance ≥1% (% of isolates)	22 (83.3)	22 (80.8)	27 (66.9)	23 (77.2)
No. genera detected in both samples (% of isolates)	20 (31.7%)		29 (33%)	
No. species detected in both samples (% of isolates)	31 (20.4%)		39 (19.9%)	
Shannon Index	3.357	3.47	3.996	3.696
Simpson Index	0.94	0.94	0.96	0.95

### Culture-Dependent Analysis of 1,500 Isolates

In order to capture a larger fraction of the high biodiversity present in bulk tank milk of these two farms, a second in-depth aerobic cultivation approach was undertaken (1,500 isolates per sample). Milk of farm A (sample 3, one milking) was collected 26 months later than sample 1, that of farm B (sample 4, two milkings) 18 months later than sample 2. Sample 3 had a TAC of 4.3 log cfu/mL and sample 4 contained 4.4 log cfu/mL (Table 1). It turned out that sample 4 comprised a high fraction of yeasts (27% of isolates) mainly belonging to the genera *Candida* and *Yarrowia*. After eliminating these from the data set, 1,102 bacterial isolates remained.

**FIGURE 2:**
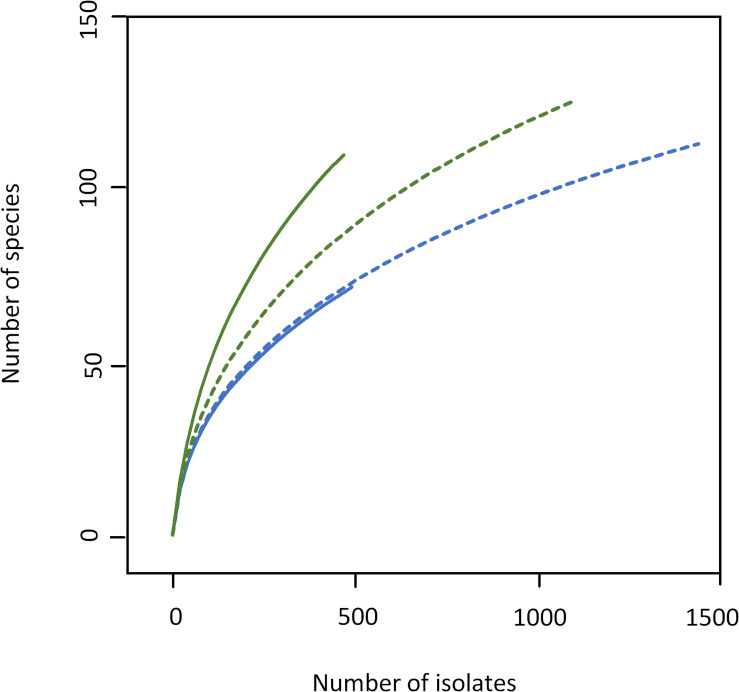
Rarefaction analysis of bulk tank milk microbiota of farm A (blue) and B (green) analyzed by cultivation. Solid lines, samples 1 and 2 (500 isolates); dotted lines, samples 3 (1,500 isolates), and 4 (1,100 isolates).

As expected, the increased sample size covered a higher biodiversity (Figure 2 and Supplementary Tables S3, S4). Samples 1 and 3 from farm A showed a nearly identical slope in rarefaction analysis (Figure 2), but 58% more species were detected in sample 3 because of the higher depth of analysis. The slope of sample 4 from farm B (1,100 colonies) was higher than for samples from farm A indicating a higher biodiversity, which is also expressed by species richness as well as Shannon and Simpson index (Table 1). However, the slope of sample 4 (only bacterial isolates, no yeast) was considerably lower than that of sample 2 (500 colonies). Although the number of isolates identified was more than doubled, only 14% more species were detected. While sample 3 was dominated by staphylococci as already observed for sample 1 (Supplementary Table S3), a shift in the dominating genera in sample 4 was recognized (Supplementary Table S4). Beside the highly abundant yeasts (data not shown), *Microbacterium* was predominant (25.6% of isolates), whereas it represented only 5.2% in sample 2. Further genera with high relative abundance in both samples were *Corynebacterium*, *Streptococcus*, and *Aerococcus*. *Pseudoxanthomonas* and *Stenotrophomonas* were detected as the most prevalent Gram-negative genera.

As the number of species with a relative abundance >1% remained constant (farm A) or even dropped slightly (farm B, Table 1) the increase in biodiversity was exclusively found among the rare species with a relative abundance <1%. Accordingly, the Simpson index did not increase with the larger sampling depth and rarefaction curves still display considerable slope (Figure 2), indicating that much more individuals need to be identified in order to better cover the detectable biodiversity.

### Molecular Analysis Based on 16S rRNA Gene Amplicon Sequencing

**FIGURE 3:**
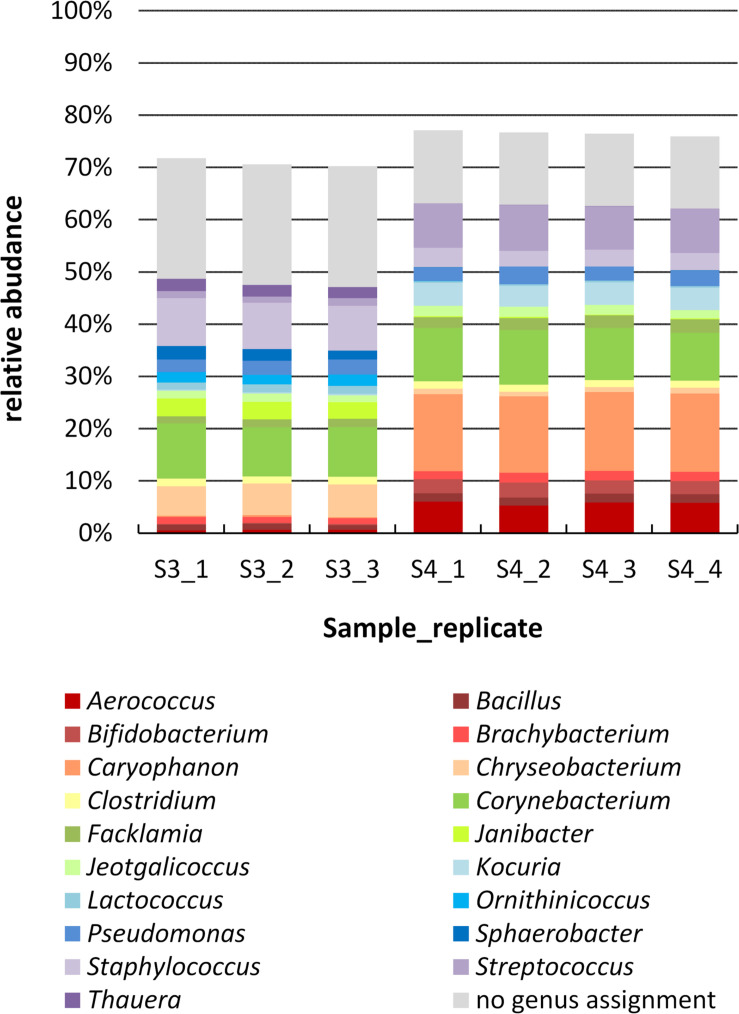
Genus level composition of samples 3 and 4 determined by amplicon sequencing. The DNA extract of each sample was analyzed by 4 PCR replicates (one of sample 3 failed in sequencing), which are shown separately. Genera are sorted in alphabetical order (bottum-up), reads with no genus assignment are shown at the very top in gray. For clarity, only genera with a relative abundance >1.5% in at least one replicate are listed; missing fractions to 100% are genera with a relative abundance <1.5%.

To study the biodiversity of microbial communities in milk samples 3 and 4 more comprehensively, a cultivation-independent approach was also followed. In order to test for reproducibility of the PCR-based library preparation, four replicates of each sample (same DNA extraction) were analyzed, each replicate consisting of eight single PCR reactions pooled together. For sample 4 (farm B), all four replicates worked and yielded a similar number of reads; for sample 3 (farm A) one replicate failed in sequencing. After data pre-processing, 30,000 high-quality reads were obtained for sample 3 and nearly 42,000 reads for sample 4. The reproducibility was very high and only minor variations were observed at the compositional level between different replicates of the same sample (Figure 3), demonstrating the robustness of the analysis.

The two farm samples displayed a similar biodiversity, both concerning the number of OTUs and genera identified (Table 2). As many as 144 genera and 75% of the 624 OTUs detected occurred in both samples. A considerable number of reads (33% in sample 3 and 21% in sample 4) belonging to 287 OTUS were not assigned to a genus using the RDP classifier. Manual identification of OTUs using EzBiocloud was able to classify nearly a quarter of those (69 OTUs). Nonetheless, 23 and 14% of reads were identified only to the level of phylum, order or family (Figure 3).

**TABLE 2:** Comparison of microbial biodiversity of samples 3 and 4 as detected by 16S rRNA amplicon sequencing and cultivation.

	Sample 3		Sample 4	
		
	Amplicons	Isolates	Amplicons	Isolates
Reads/isolates	29,950	1,475	41,609	1,102
No. OTUs/species	555	112	535	125
No. families	87	33	83	41
No. families in common	28		36	
No. genera	162	47	166	60
No. genera in common	34		46	
No. genera with relative abundance ≥1% (% of reads or isolates)	20 (52.6)	14 (91.2)	17 (65.9)	21 (90.7)
No. OTUs/species with relative abundance ≥1% (% of reads or isolates)	18 (39.5)	22 (80.8)	19 (55.8)	23 (77.2)
Shannon Index	5.78	3.47	4.54	3.70
Simpson Index	0.999	0.94	0.999	0.95

Although the microbiota resembled each other in terms of overall taxa occurrence, there were marked discrepancies in relative abundances, already at the phylum level. The most abundant genus in sample 4 was *Caryophanon* (15%) belonging to the *Firmicutes* that was only rarely detected in sample 3 (0.3%). Sample 3, in contrast, contained higher fractions of *Chryseobacterium* (*Bacteroidetes*), *Janibacter* (*Actinobacteria*), and *Staphylococcus* (*Firmicutes*) whereas sample 4 harbored more *Kocuria* (*Actinobacteria*) as well as *Aerococcus* and *Streptococcus* (*Firmicutes*). Altogether, sample 3 was characterized by a higher fraction of *Bacteroidetes*, whereas sample 4 contained more *Firmicutes.* Still, some abundant genera such as *Bacillus*, *Brachybacterium*, *Clostridium*, *Corynebacterium*, *Facklamia*, *Jeotgalicoccus*, and *Pseudomonas* occurred in similar fractions in both samples (Figure 3).

### Comparison of Culture- and Sequencing-Based Biodiversity

**FIGURE 4:**
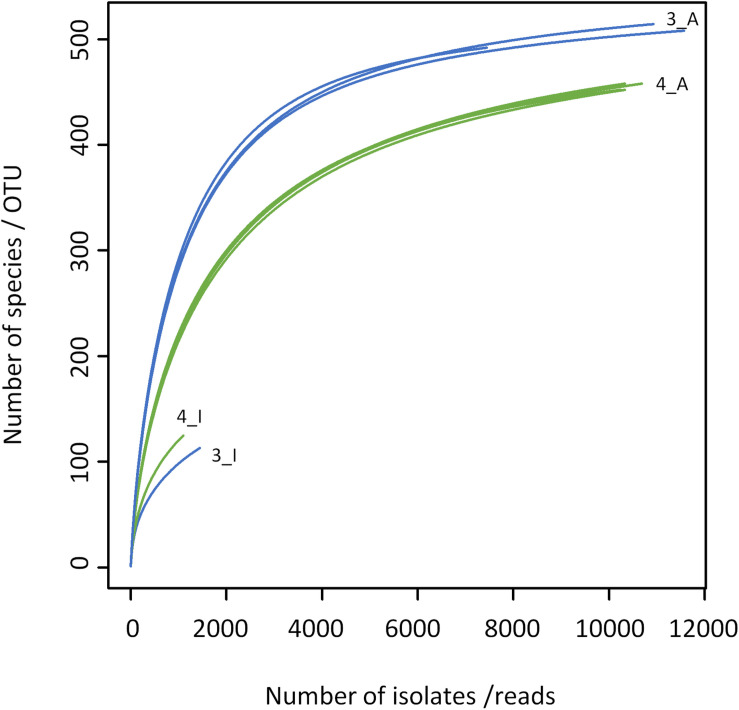
Rarefaction analysis of microbiota of bulk tank milk samples 3 (blue) and 4 (green) analyzed by a cultivation-based isolation (3_I, 4_I) or amplicon sequencing (3_A, 4_A). Individual PCR replicates are shown for the amplicon approach.

**FIGURE 5:**
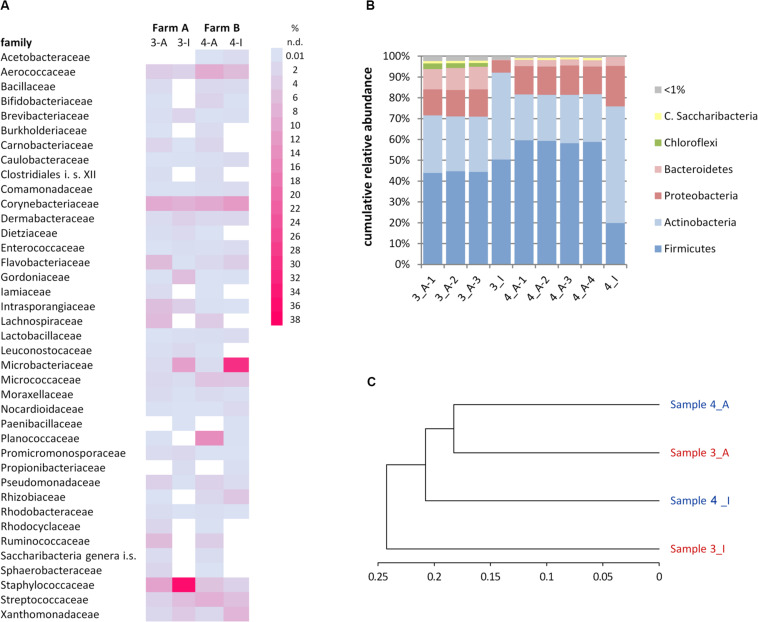
Comparison of microbiota in samples 3 (farm A) and 4 (farm B) as detected by the cultivation (3-I, 4-I) and molecular (3-A, 4-A) approaches. **(A)** Relative abundance of families. Only families >1% in at least one analysis are listed. i.s., incertae sedis; n.d., not detected. **(B)** Relative abundance of phyla. C., Candidatus. **(C)** Phylogram based on the Ward’s minimum variance method showing the hierarchical clustering of samples. Genus-level data were used to calculate distances; only the 57 genera detected at least once by both the cultivation-dependent and –independent analyses were included.

The results obtained by the two different approaches exhibit many discrepancies (Figures 4, 5 and Table 2). Amplicon sequencing detected a higher biodiversity, as 4–5 times more OTUs than species, 2.8–3.4 times more genera and 2–3 times more families were identified. This is also reflected by higher Shannon and Simpson indices (Table 2). However, there were only few highly abundant genera (>1%) and this favored the detection of a large biodiversity. Additionally, they sum up to a relatively small fraction of reads (53 and 66%, respectively), whereas in the cultivation-dependent approach they represented 90% of isolates. This allows for the discovery of many more minor genera and agrees with the trend already observed for the cultivation-dependent analysis of samples 2 and 4. In addition, sample 3 exhibited a higher biodiversity than sample 4 in the amplicon-based approach, although fewer reads were obtained (Table 2). This clearly contradicts the results obtained by the cultivation-dependent analysis. Rarefaction curves of amplicon analyses flattened for both samples indicating that the largest fraction of biodiversity has been detected within the approximately 10,000 reads of one PCR replicate (Figure 4). However, combining the three or four replicate PCRs for each sample still increased the biodiversity by 10% for sample 3 (3 replicates) and 17% for sample 4 (4 replicates).

Many genera not detected using the culture-dependent approach belonged to families that grow anaerobically or are not culturable using TSA under aerobic conditions such as *Lachnospiraceae*, *Rhodocyclaceae*, *Ruminococcaceae*, Saccharibacteria *incertae sedis*, *Sphaerobacteraceae*, or *Clostridiales incertae sedis* (Supplementary Table S5). These missing taxa lead to the much lower slope of rarefaction curves of the culture-based analysis (Figure 4). The biggest discrepancy was observed in sample 4, where *Caryophanon* belonging to the *Planococcaceae* was detected as the most abundant genus by amplicon sequencing accounting for almost 15% of reads, but was not isolated a single time in the culture-dependent analysis due to its fastidious growth requirements. However, although the sequencing approach revealed a largely higher biodiversity, there were still 24 genera not detected, but found by culturing. Two thirds of these belonged to the *Actinobacteria* and 21% to the *Firmicutes* (Supplementary Table S6). Eight genera belonged to six families that were not identified by amplicon sequencing. *Jonesiaceae* and *Morganellaceae* were detected exclusively by culturing in sample 3, *Mycobacteriaceae* and *Tsukamurellaceae* only in sample 4 and in both samples *Paenibacillaceae* and *Propionibacteriaceae* were overlooked in the molecular analysis. In summary, none of the approaches alone is able to cover and detect the full biodiversity present in the samples.

It was furthermore observed that the relative abundance of some families is massively distorted depending on the type of analysis (Figure 5A). *Microbacteriaceae* and *Xanthomonadaceae* were considerably underrepresented in the amplicon approach in both samples as well as *Staphylococcaceae* or *Gordoniaceae* in sample 3, whereas *Flavobacteriaceae* or *Intrasporangiaceae* were overrepresented. The misestimation of *Microbacteriaceae* and *Gordoniaceae* (*Actinobacteria*) as well as *Staphylococcaceae* and *Planococcaceae* (*Firmicutes*) lead to substantial differences observed already at the phylum level (Figure 5B). To correlate the similarities between the two samples and both techniques and to visualize methodology-driven discrepancies, a phylogram was calculated (Figure 5C). It was based on those 57 genera that have been determined at least once using each approach in order to emphasize the distribution of relative abundances rather than the differences in biodiversity. It thus included 50 and 59% of reads of sample 3 and 4, respectively, the remaining OTUs were not considered. Data obtained by amplicon sequencing showed the highest similarities and the culture-based analyses were more distantly related. Differences introduced by methodology, thus, exceeded sample-specific differences. But besides all discrepancies observed, many of those families identified in a sample by both techniques were captured with relatively similar fractions (Figure 5A and Supplementary Table S5) revealing substantial overlap in results between the two techniques.

## Discussion

The four milk samples analyzed in this study contained a microbiota typical for fresh bovine raw milk dominated by Gram-positive bacteria (Delbes et al., 2007; Fricker et al., 2011; Curone et al., 2018; Lima et al., 2018), although the bulk tank milk of samples 1 and 2 had been stored at the farm for 3 or 2 days before collection (combining six or four milkings, respectively), which is thought to favor the growth of Gram-negative psychrotolerant genera such as *Pseudomonas*. The major phyla detected were *Firmicutes*, *Actinobacteria*, *Proteobacteria*, and *Bacteroidetes* as already found for bulk tank milk or milk from single healthy animals in other studies (Ganda et al., 2016; Rodrigues et al., 2017; Fretin et al., 2018; Lima et al., 2018). Kable et al. (2016) analyzed the microbiome of almost 900 samples of tanker trucks covering 200 farms in California and found 29 taxa belonging to the core microbiome present in all samples. 18 were identified down to genus level, the remaining eleven only to the family level. Of the 18 taxa identified at genus level four (*Butyrivibrio*, *Yaniella*, *Mycoplasma*, 5-7N15) were absent in samples 3 and 4 in this study, indicating that regional characteristics may influence the milk microbiota and detected core microbiomes will be significantly affected by various factors, some of which are probably still unknown. Numerous studies highlighted the influence of farm practices, herd management, milking hygiene (Verdier-Metz et al., 2009; Vacheyrou et al., 2011; Mallet et al., 2012; Miller et al., 2015; Fretin et al., 2018; Metzger et al., 2018), season (Doyle et al., 2017b; Li et al., 2018), lactation stage (Doyle et al., 2017a; Curone et al., 2018) or breed (Curone et al., 2018) on the bovine milk microbiome, but many more studies are needed to elucidate the complex relationships between different factors associated with milk production and milk microbiota. Because of this multifactorial influence, outcomes of many studies regarding species composition differ largely. In the present study, we found *Staphylococcus*, *Corynebacterium, Caryophanon*, or *Streptococcus* as leading genera in the amplicon approach, whereas Li et al. (2018) found the four genera *Bacillus*, *Acinetobacter*, *Pseudomonas*, and *Lactococcus* making up more than half of all reads.

With the advance of next-generation sequencing techniques, huge data sets can be generated and very comprehensive studies have become feasible. To be able to relate these new data to outcomes of former studies based on the cultivation and identification of isolates, comparative analyses are necessary. To ensure comparability, an adequate depth of culture-based analysis is required, which is laborious. In the first part of the present study, a large biodiversity could be detected in bulk tank milk samples using a culture-dependent analysis. Although only the aerobic mesophilic bacteria were analyzed, more than 100 species were identified out of 500 isolates in sample 2. For samples 3 and 4, dedicated to analysis by both culture-dependent and –independent techniques, the number of isolates was increased to 1,500, restricting the analysis to the aerobic bacteria growing on TSA in order to strengthen the validity for taxa related to food quality and safety and to retain a high sampling depth for the chosen culture condition. In contrast, 16S rRNA gene amplicon sequencing detects prokaryotic taxa regardless of growth requirements and, in addition, was based on a larger sample volume. For DNA extraction 150 mL milk were centrifuged to concentrate bacterial cells. In the culture dependent approach, however, the 1,500 colonies picked from agar plates were contained in <0.1 mL of sample. This difference is reflected by the three times higher number of genera identified among sequence reads vs. isolates. As the sample preparation did not include a step using propidium monoazide (PMA) to block DNA from dead cells, part of the detected biodiversity may not belong to the viable fraction of the microbiota. However, Kable et al. (2019) found equivalent cell numbers as well as no detectable differences in biodiversity between raw milk samples with and without PMA treatment. We therefore conclude that raw milk contains only a minor fraction of dead cells which does not significantly contribute to the biodiversity detected.

Nevertheless, species richness in sequencing data may be overestimated, as 54 of all OTUs (8.6%) were classified only to the phylum level or above. These unknown taxa detected may indeed exist, or may represent sequencing artifacts, which can never be completely removed during processing (Edgar, 2013; Allali et al., 2017). Increasing threshold levels for relative abundance filtering would contribute to diminishing this problem, but would also exclude real taxa. In the present study, a relative abundance threshold of 0.1% would have detected 29 genera less than the 0.05% filter threshold applied. This equals 16% of all genera detected. Six of those were also detected by culturing and, thus, are evidentially no artifacts. Diakite et al. (2019) reported similar findings for the analysis of fecal microbiota. They detected 27 bacterial species using cultivation that were represented by less than five sequence reads at an average sequencing depth of approximately 100,000 reads per sample (equaling < 0.005% of reads). Relative abundance filtering should therefore be combined with an additional parameter such as identification to a certain taxonomic level to improve efficiency of filtering artifacts.

To the contrary, 24 genera were not detected by sequencing, but found using cultivation. The majority of these culture-specific taxa were of very low relative abundance and often represented by only one isolate. However, some occurred more often such as *Agromyces*, *Citricoccus*, *Jonesia*, *Luteococcus*, *Tessaracoccus*, and *Mycobacterium*, representing 0.35–0.75% of all isolates in the respective sample. The high sensitivity of culture-based approaches concerning the detection of species has also been shown by other studies. Diakite et al. (2019) applied a large-scale culture approach for fecal samples using 58 different conditions and detected 494 species while sequencing was able to uncover merely 362 species (73%). Analyzing the taxonomic assignment of OTUs in the present study revealed that *Actinobacteria* were overrepresented among those 24 genera not detected (66%) while they made up only 44% of genera found by culturing and 22% in amplicon sequencing. Three of the 24 genera (*Brevibacillus*, *Paenibacillus*, and *Rummeliibacillus*) belonged to spore forming bacteria and DNA from endospores will remain undetected in molecular analyses.

It is very likely that missing or underestimating genera is at least partially methodology driven, choice of primers and DNA extraction from Gram-positive bacteria probably being the most critical steps (Klindworth et al., 2013; Parada et al., 2016; Panek et al., 2018; Vaidya et al., 2018). We found that four of the 24 taxa that were not detected (among them *Luteococcus* and *Tessaracoccus*) as well as *Microbacterium*, the most underestimated genus in the amplicon-based approach, exhibited a mismatch in the reverse primer pairing. All five genera belonged to the families *Microbacteriaceae* or *Propionibacteriaceae* (Supplementary Table S6) and showed the same substitution at the third position of the 785R primer (C instead of A). However, all other 20 undetected genera as well as *Gordonia* and *Staphylococcus*, that were greatly underestimated, showed a perfect primer match.

Numerous studies confirm that the method of DNA extraction leads to shifts in the microbiome most probably due to differential lysis of various taxa (Biesbroek et al., 2012; Hart et al., 2015; Panek et al., 2018; Vaidya et al., 2018). *Actinobacteria* are particularly challenging in cell lysis due to their strong cell walls. In accordance to the present study, Fretin et al. (2018) also missed many *Actinobacteria* and particularly *Microbacterium* species when comparing culture-dependent and -independent analyses of raw milk samples. Similar findings were observed by Treven et al. (2019) for human milk microbiota. Besides, Laursen et al. (2017) reported a negative correlation of detected relative abundance with genomic G+C content, which was largely diminished by elongating the denaturation step in the PCR from 30 to 120 seconds. In the present study, the denaturation step lasted 30 s and could also be a reason why *Microbacterium* and other genera belonging to the *Actinobacteria* were greatly underestimated.

Another reason why some taxa were missed may be their resistance to lytic enzymes that renders them difficult to lyse. This is well known for many species e.g., belonging to the Firmicutes such as staphylococci and often originates from modifications of the peptidoglycan structures (Davis and Weiser, 2011). The PathoProof Mastitis Kit used for DNA extraction in the present study does not include a bead-beating step, which strengthens the dependency on enzymatic lysis. In the end, there are multiple reasons for discrepancies between cultivation-dependent and –independent analyses, which may, however, be at least partially overcome when eliminating possible sources of deficiencies in the sample preparation steps by appropriately adaptating the protocol for DNA extraction and library preparation, e.g., by adding a bead-beating step.

Species identified by culture covered between 20 and 24% of OTUs detected in this study and represented only a minor fraction of biodiversity. During the last years, several studies demonstrated that the cultured fraction of microbiota can be substantially improved by applying numerous culture media and conditions in parallel and increasing sampling effort, referred to as culturomics (Lagier et al., 2012) or culture-enriched molecular profiling (Lau et al., 2016) and may even largely exceed the number of OTUs detected by culture-independent sequencing (Lau et al., 2016; Diakite et al., 2019). Besides the complementary nature of the two approaches in estimating biodiversity, capturing unknown species by culture is necessary to allow for species characterizations, to reveal possible interactions in the microbiome or to determine implications for food processing (in case of food microbiomes). The core microbiome of bovine milk analyzed by Kable et al. (2016) using amplicon sequencing included almost 40% of unclassified taxa. However, the relevance of many of these remains unclear. Our study is the first using an intense and in-depth culture-based analysis of milk microbiota. Although only one common cultivation condition was applied, numerous potential novel species and genera have been detected. Across all four samples between 20 and 30% of species are hitherto unknown and they summed up to 15.5 and 23% of all isolates identified in samples 1 and 2, respectively. Culturing is therefore still a precious and essential effort in microbial taxonomy and ecology.

## Conclusion

Raw milk microbiota contain a high biodiversity that can be relatively well covered by classical aerobic cultivation as long as hundreds of isolates are analyzed. Cultivation is valuable to unravel and characterize unknown taxa and make a deposit in culture collections to guarantee broad availability. Amplicon sequencing, in contrast, provides a higher resolution of analysis, but may also include the analysis of artifacts due to methodological bias. Distortion of relative abundance estimation of single genera was observed in the present study, which increases the risk of misinterpretations. Hence, protocols for library preparation need to be optimized, with special attention to cell lysis of Gram-positive bacteria and PCR conditions, and bioinformatic processing must be carefully thought through. As culture-based analysis of the raw milk microbiome including anaerobic and fastidious species is laborious, further comprehensive studies will require both technical improvements to enhance throughput and the use of molecular techniques.

## Data Availability Statement

The datasets presented in this study can be found in online repositories. The amplicon sequences of this study are available in the Sequence Read Archive under Bioproject PRJNA622551.

## Author Contributions

FB and ED carried out experiments in the lab. FB performed identification of Sanger and amplicon sequences. TC supported evaluation of amplicon data and helped with manuscript writing. MW and SS designed the study. SS corrected the manuscript. MW was responsible for data evaluation and wrote the manuscript. All authors contributed to the article and approved the submitted version.

## Conflict of Interest

FB was employed by Milchprüfring Baden-Württemberg e.V. during the course of the study. The remaining authors declare that the research was conducted in the absence of any commercial or financial relationships that could be construed as a potential conflict of interest.
