# Supplementary material for: Complementary Use of Cultivation and High-Throughput Amplicon Sequencing Reveals High Biodiversity Within Raw Milk Microbiota
Source: Front Microbiol. 2020 Jul 9;11:1557. doi: 10.3389/fmicb.2020.01557 (PMC7365021; doi:10.3389/fmicb.2020.01557)
Supplement: Supplementary file 1 [file Data_Sheet_1.PDF]

Supplementary Table S1

Relative abundance of species detected via cultivation in sample 1, farm A. For potential novel species (sp. nov.) or novel genera (gen. nov) the most similar species or genus is given in brackets.

**Species**	**relative abundance**
Staphylococcus equorum	13,5%
Staphylococcus vitulinus	12,4%
Corynebacterium xerosis	8,8%
Janibacter anophelis	7,2%
Brachybacterium sp. nov (phenoliresistens)	5,4%
Corynebacterium casei	3,6%
Acinetobacter sp. nov. (haemolyticus)	3,4%
Isoptericola halotolerans	3,4%
Dietzia aerolata	2,6%
Staphylococcus haemolyticus	2,6%
Corynebacterium camporealensis	2,4%
Brochothrix thermosphacta	2,2%
Corynebacterium humireducens	2,2%
Leuconostoc mesenteroides	2,2%
Streptococcus uberis	2,2%
Aerococcus suis	1,8%
Chryseobacterium haifense	1,8%
Staphylococcus xylosus	1,4%
Brachybacterium sp. nov. (paraconglomeratum)	1,0%
Corynebacterium stationis	1,0%
Microbacterium maritypicum/oxydans	1,0%
Ornithinimicrobium murale	1,0%
Arthrobacter sp. nov (kerguelensis)	0,8%
Brachybacterium nesterenkovii	0,8%
Brevibacterium senegalense	0,8%
Kocuria salsicia	0,8%
Luteimonas sp. nov. (terricola)	0,8%
Brachybacterium saurashtrense	0,6%
Facklamia tabacinasalis	0,6%
Mesorhizobium sp. nov. (tamadayense)	0,6%
Bacillus kochii	0,4%
Brachybacterium paraconglomeratum	0,4%
Chryseobacterium anthropi	0,4%
Chryseobacterium bovis	0,4%
Corynebacterium efficiens	0,4%
Corynebacterium sp. nov. (pilosum)	0,4%
gen. nov. (Facklamia)	0,4%
Jeotgalicoccus psychrophilus	0,4%
Staphylococcus chromogenes	0,4%
Staphylococcus sciuri ssp. carnaticus	0,4%
Actinotalea ferrariae	0,2%
Aerococcus urinaeequi	0,2%
Arthrobacter sp. nov. (globiformis)	0,2%
Brachybacterium ginsengisoli	0,2%
Brevibacterium epidermidis	0,2%

Species	relative abundance
Brevibacterium luteolum	0,2%
Brevibacterium yomogidense	0,2%
Cellulomonas phragmiteti	0,2%
Corynebacterium sp. nov. (lubricantis)	0,2%
Corynebacterium sp. nov. (efficiens)	0,2%
Corynebacterium sp. nov. (lipophiloflarum)	0,2%
Dietzia alimentaria	0,2%
Dietzia auranziaca	0,2%
Facklamia miroungae	0,2%
gen. nov. (Eremococcus)	0,2%
Georgenia sp. nov. (thermotolerans)	0,2%
Globicatella sulfidifaciens	0,2%
Jeotgalibaca sp. nov. (dankookensis)	0,2%
Lactococcus laudensis	0,2%
Mesorhizobium sp. nov. (thiogangeticum)	0,2%
Microbacterium sp. nov. (awajiense)	0,2%
Microbacterium sp. nov. (gubbeenense)	0,2%
Micrococcus sp. nov. (terreus)	0,2%
Moraxella osloensis	0,2%
Myceligenerans sp. nov. (halotolerans)	0,2%
Nesterenkonia lacusekhoensis	0,2%
Nosocomiicoccus ampullae	0,2%
Pseudoclavibacter helvolus	0,2%
Staphylococcus hominis	0,2%
Staphylococcus sp. nov. (xylosus)	0,2%
Streptococcus dysgalactiae	0,2%
not identified	0,6%

Supplementary Table S2

Relative abundance of species detected via cultivation in sample 2, farm B. For potential novel species (sp. nov.) or novel genera (gen. nov) the most similar species or genus is given in brackets.

Species	relative abundance
Corynebacterium xerosis	10,4%
Knoellia sp. nov. (flava/sinensis)	7,8%
Streptococcus dysgalactiae ssp. dsygalactiae	4,8%
Chryseobacterium sp. nov. (treverense)	4,2%
Kocuria atrinae	3,2%
Rothia endophytica	3,2%
Streptococcus uberis	3,0%
Aerococcus urinaeequi	2,6%
Janibacter limosus	2,4%
Kocuria carniphila	2,4%
Chryseobacterium haifense	2,2%
Corynebacterium camporealensis	2,2%
Microbacterium lacticum	2,0%
Microbacterium maritypicum/oxydans	2,0%
Chryseobacterium bovis	1,8%
Lactococcus raffinolactis	1,8%
Propionibacterium jensenii	1,6%
Corynebacterium flavescens	1,4%
Luteococcus sp. nov. (sediminum)	1,4%
Corynebacterium frankenforstense	1,2%
Enterococcus faecalis	1,2%
Moraxella osloensis	1,2%
Ottowia sp. nov. (beijingensis)	1,2%
Corynebacterium variabile	1,0%
Dietzia alimentaria	1,0%
Luteimonas sp. nov. (aestuarii)	1,0%
Thermomonas sp. nov. (brevis)	1,0%
Aerococcus suis	0,8%
Brevundimonas intermedia	0,8%
Corynebacterium sp. nov. (epidermidicanis)	0,8%
gen. nov. (Propioniciclava)	0,8%
Pseudomonas lundensis	0,8%
Psychrobacter sanguinis	0,8%
Brevibacterium pityocampae	0,6%
Brevundimonas lenta	0,6%
Kocuria salsicia	0,6%
Lactococcus laudensis	0,6%
Leuconostoc lactis	0,6%
Microbacterium flavescens	0,6%
Nocardioides daeguensis	0,6%
Sphingobacterium daejeonense	0,6%

**Species**	**relative abundance**
Staphylococcus haemolyticus	0,6%
Acinetobacter parvus	0,4%
Acinetobacter sp. nov. (haemolyticus)	0,4%
Brachybacterium nesterenkovii	0,4%
Brevibacterium celere	0,4%
Chryseobacterium sp. nov. (aahli)	0,4%
Chryseobacterium ureilyticum	0,4%
Corynebacterium amycolatum	0,4%
Corynebacterium humireducens	0,4%
Facklamia tabacinasalis	0,4%
gen. nov. (Auraticoccus)	0,4%
Gordonia bronchialis	0,4%
Jeotgalicoccus psychrophilus	0,4%
Knoellia sp. nov. (subterranea)	0,4%
Lactobacillus paracasei	0,4%
Leucobacter iarius	0,4%
Luteococcus japonicus	0,4%
Microbacterium yannicii	0,4%
Staphylococcus chromogenes	0,4%
Acinetobacter lwoffii	0,2%
Acinetobacter breziniae	0,2%
Acinetobacter johnsonii	0,2%
Acinetobacter sp. nov. (harbinensis)	0,2%
Aerococcus sp. nov. (suis)	0,2%
Aeromicrobium flavum	0,2%
Agrococcus jenensis	0,2%
Arthrobacter sp. nov. (soli)	0,2%
Bosea vestrisii	0,2%
Brachybacterium muris	0,2%
Branchiibius hedensis	0,2%
Brevibacterium luteolum	0,2%
Brevibacterium senegalense	0,2%
Brevibacterium sp. nov. (daeguensis)	0,2%
Brevundimonas mediterranea	0,2%
Brevundimonas staleyi	0,2%
Chryseobacterium hominis	0,2%
Chryseobacterium sp. nov. (haifense)	0,2%
Chryseobacterium sp. nov. (limigenitum)	0,2%
Comamonas koreensis	0,2%
Corynebacterium sp. nov. (doosanense)	0,2%
Corynebacterium bovis	0,2%
Corynebacterium confusum	0,2%
Corynebacterium sp. nov. (pilosum)	0,2%
Corynebacterium ulceribovis	0,2%
Corynebacterium urealyticum	0,2%
Enterobacter ludwigii	0,2%
gen. nov. (Eremococcus)	0,2%

Species	relative abundance
gen. nov. (Modestobacter)	0,2%
gen. nov. (Sphingobacterium)	0,2%
Globicatella sulfidifaciens	0,2%
Gordonia aichiensis	0,2%
Kurthia gibsonii	0,2%
Lactococcus lactis ssp. lactis	0,2%
Mesorhizobium sp. nov. (robiniae)	0,2%
Microbacterium xylanilyticum	0,2%
Nosocomiicoccus ampullae	0,2%
Prolinoborus sp. nov. (fasciculus)	0,2%
Pseudoclavibacter chungangensis	0,2%
Pseudomonas sp. nov. (alcaligenes)	0,2%
Pseudoxanthomonas mexicana	0,2%
Psychrobacter faecalis	0,2%
Rhzorhapis sp. nov. (suberifaciens)	0,2%
Rothia nasimurium	0,2%
Shinella zoogloeoides	0,2%
Sphingopyxis bauzanensis	0,2%
Staphylococcus epidermidis	0,2%
Staphylococcus hominis	0,2%
Trueperella sp. nov. (pyogenes)	0,2%
Weissella thailandensis	0,2%
not identified	3,6%

Supplementary Table S3

Relative abundance of species detected via cultivation in sample 3, farm A.
For potential novel species (sp. nov.) or novel genera (gen. nov) the most similar species or genus is given in brackets.

**Species**	**relative abundance**
Staphylococcus vitulinus	12,9%
Staphylococcus haemolyticus	11,8%
Microbacterium lacticum	9,9%
Gordonia caeni	6,2%
Pseudoxanthomonas mexicana	4,4%
Corynebacterium xerosis	4,3%
Janibacter anophelis	3,2%
Staphylococcus xylosus	3,0%
Staphylococcus sciuri	2,7%
Corynebacterium stationis/casei	2,4%
Streptococcus uberis	2,3%
Streptococcus pluranimalium	2,1%
Staphylococcus spec. nov. (hyicus)	2,0%
Brevibacterium epidermidis/iodinum	2,0%
Aerococcus urinaeequi/viridans	1,8%
Isoptericola halotolerans	1,8%
Brachybacterium conglomeratum/paraconglomeratum	1,4%
Leuconostoc mesenteroides	1,4%
Staphylococcus aureus	1,4%
Staphylococcus equorum	1,4%
Brachybacterium spec. nov. (paraconglomeratum)	1,2%
Dietzia aurantiaca	1,0%
Aerococcus suis	0,9%
Staphylococcus chromogenes	0,9%
Enterococcus faecalis	0,7%
Lactococcus lactis	0,7%
Corynebacterium confusum	0,6%
Lactococcus raffinolactis	0,6%
Staphylococcus spec. nov. (haemolyticus)	0,6%
Dietzia aerolata	0,5%
Pseudoclavibacter helvolus	0,5%
Brevibacterium senegalense/yomogidense	0,5%
Staphylococcus epidermidis	0,5%
Citricoccus spec. nov. (alkalitolerans)	0,3%
Corynebacterium camporealensis	0,3%
Jonesia denitrificans	0,3%
Luteococcus japonicus	0,3%
Luteococcus sanguinis	0,3%
Corynebacterium bovis	0,3%
Lactobacillus paracasei	0,3%
Microbacterium maritypicum/oxydans	0,3%

Species	relative abundance
Staphylococcus spec. nov. (xylosus)	0,3%
Streptococcus infantarius/lutetiensis/equinus	0,3%
Brachybacterium gingsengisoli	0,2%
Corynebacterium spec. nov. (argentoratense)	0,2%
Klebsiella granulomatosis/pneumoniae	0,2%
Kocuria carniphila	0,2%
Kocuria palustris	0,2%
Lactococcus piscium	0,2%
Lactococcus plantarum	0,2%
Leuconostoc pseudomesenteroides	0,2%
Ottowia spec. nov. (beijingensis)	0,2%
Microbacterium spec.	0,2%
Pseudomonas spec.	0,1%
Agrococcus casei	0,1%
Arsenicicoccus bolidensis	0,1%
Brachybacterium spec. nov. (gingsengisoli)	0,1%
Chryseobacterium haifense	0,1%
Corynebacterium glutamicum	0,1%
Facklamia tabascinasalis	0,1%
gen nov. (Propioniciclava tarda)	0,1%
Georgenia spec. nov. (muralis)	0,1%
Gordonia alkanivorans	0,1%
Microbacterium hominis	0,1%
Microbacterium lacus	0,1%
Ottowia spec. nov. (pentelensis)	0,1%
Phenylobacterium haematophilum	0,1%
Psychrobacter pulmonis	0,1%
Rhodococcus qingshengii/jialingiae/baikanurensis	0,1%
Sphingopyxis witflariniensis	0,1%
Staphylococcus hominis	0,1%
Staphylococcus simulans	0,1%
Acinetobacter parvus	0,1%
Aerococcus spec. nov. (suis)	0,1%
Agrococcus baldri	0,1%
Arthrobacter cryoconiti	0,1%
Arthrobacter phenanthrenivrans/kerguelensis	0,1%
Arthrobacter spec. nov. (phenonthrenivorans/oryzae/subteranu	0,1%
Brachybacterium alimentarium/sacelli	0,1%
Brachybacterium spec. nov. (phenoliresistens)	0,1%
Brachybacterium spec. nov. (sacelli)	0,1%
Brevundimonas bullata	0,1%
Chryseobacterium indoltheticum	0,1%
Corynebacterium amycolatum	0,1%
Corynebacterium aurimucosum	0,1%
Corynebacterium freneyi/hansenii	0,1%
Corynebacterium tuberculostearicum	0,1%
Curtobacterium flaccumfaciens	0,1%

Species	relative abundance
gen. nov. (Aerococcus suis)	0,1%
Gordonia spec. nov. (caeni)	0,1%
Gordonia terrae	0,1%
Janibacter terrae	0,1%
Jeotgalibaca spec. nov. (dankookensis)	0,1%
Kocuria salsicia	0,1%
Lactobacillus oligofermentans	0,1%
Lactobacillus parabuchneri	0,1%
Luteococcus spec. nov. (sanguinis)	0,1%
Microbacterium gubbeenense	0,1%
Nocardioides spec. nov. (konjugensis)	0,1%
Paenibacillus amylolyticus	0,1%
Paracoccus alcaliphilus	0,1%
Plantibacter flavus	0,1%
Proteus mirabilis	0,1%
Pseudomonas aeruginosa	0,1%
Pseudomonas azotoformans/libanensis	0,1%
Rhodococcus cerastii	0,1%
Rhodococcus fascians	0,1%
Sphingobacterium deajeonaese	0,1%
Staphylococcus cohnii	0,1%
Staphylococcus spec. nov. (auricularis)	0,1%
Staphylococcus spec. nov. (equorum)	0,1%
Tessaraococcus spec. nov. (flavescens)	0,1%
not identified	0,2%

Supplementary Table S4

Relative abundance of species detected via cultivation in sample 4, farm B.
For potential novel species (sp. nov.) or novel genera (gen. nov)
the most similar species or genus is given in brackets.

**Species**	**relative abundance**
Microbacterium yannicii	13,6%
Microbacterium lacticum/saccharophilum	9,6%
Stenotrophomonas maltophilia	7,0%
Corynebacterium xerosis/freneyi	5,4%
Aerococcus urinaeequi/viridans	4,5%
Kocuria salsicia	3,8%
Streptococcus uberis	3,8%
Chryseobacterium ureilyticum	3,4%
Shinella zoogloeoides	3,4%
Corynebacterium frankenforstense	3,3%
Pseudoclavibacter sp. nov. (caeni)	3,1%
Streptococcus dysgalactiae ssp dysgalactiae	2,0%
Aerococcus suis	1,7%
Brachybacterium sp. nov. (sacelli)	1,5%
Corynebacterium variabile	1,5%
Leucobacter komagatae/aridicollis	1,5%
Brevundimonas vesicularis/nasdae	1,3%
Microbacterium maritypicum/oxydans	1,3%
Acidovorax wautersii	1,2%
Bacillus licheniformis/aerius	1,2%
Acetobacter orientalis	1,1%
Rhizobium radiobacter	1,1%
Nocardioides sp. nov. (kongjuensis)	1,0%
Corynebacterium amycolatum	0,9%
Enterococcus faecalis	0,8%
Agromyces mediolanus/soli	0,6%
Corynebacterium confusum	0,6%
Pseudomonas aeruginosa	0,6%
Staphylococcus hominis	0,6%
Stenotrophomonas terrae	0,6%
Dermacoccus sp. nov. (barathri/abyssi)	0,5%
Kocuria atrinae	0,5%
Pseudoclavibacter helvolus	0,5%
Staphylococcus cohnii	0,5%
Tesseracoccus sp. nov. (flavescens)	0,5%
Bosea robiniae	0,5%
Microbacterium sp. nov. (dextranolyticum)	0,5%
Corynebacterium falsenii	0,4%
Jeotgalicoccus marinus	0,4%
Lactobacillus parabuchneri	0,4%
Lactobacillus paracasei	0,4%

Species	relative abundance
Mycobacterium abscessus/chelonae	0,4%
Nocardioides daeguensis	0,4%
Shinella granuli	0,4%
Acinetobacter bereziniae/guillouiae	0,3%
Bifidobacterium psychraerophilum	0,3%
Brachybacterium nesterenkovii	0,3%
Corynebacterium humireducens	0,3%
Globicatella sulfidifaciens	0,3%
Microbacterium dextranolyticum/natoriense	0,3%
Microbacterium hominis	0,3%
Pseudoclavibacter chungangensis	0,3%
Staphylococcus haemolyticus	0,3%
Staphylococcus sciuri	0,3%
Stenotrophomonas nitritireducens	0,3%
Acinetobacter johnsonii	0,2%
Aerococcus sp. nov. (suis)	0,2%
Aquamicrobium lusatiense	0,2%
Bacillus sonorensis	0,2%
Corynebacterium bovis	0,2%
Enterococcus malodoratus	0,2%
Facklamia hominis	0,2%
Flavobacterium sp. nov. (mizutaii)	0,2%
gen. nov. (Corynebacterium doosanense)	0,2%
Gordonia sputi	0,2%
Kocuria palustris	0,2%
Lactococcus garvieae	0,2%
Ochrobactrum rhizosphaerae	0,2%
Ochrobactrum sp. nov. (anthropi/cytisi/lupini)	0,2%
Ochrobactrum thiophenivorans/cytisi	0,2%
Patulibacter ginsengiterrae	0,2%
Pseudomonas alcaligenes	0,2%
Pseudomonas peli	0,2%
Pseudomonas sp. nov. (alcaligenes)	0,2%
Sphingobacterium daejoenense	0,2%
Staphylococcus epidermidis	0,2%
Staphylococcus jettensis	0,2%
Acinetobacter junii	0,1%
Actinomyces sp. nov. (odontolyticus)	0,1%
Arthrobacter sp. nov. (nicotianae/protophormiae)	0,1%
Arthrobacter woluwensis	0,1%
Brachybacterium muris	0,1%
Brachybacterium squillarum	0,1%
Brevibacillus agri	0,1%
Brevibacillus inovocatus/brevis	0,1%
Brevibacterium epidermidis	0,1%
Brevibacterium luteolum	0,1%
Brevibacterium senegalense	0,1%

Species	relative abundance
Brevibacterium yomogidense	0,1%
Cellulosimicrobium funkei	0,1%
Corynebacterium doosanense	0,1%
Corynebacterium sp. nov. (casei)	0,1%
Dermacoccus nishinomiyaensis	0,1%
Devosia riboflavina	0,1%
Enterococcus viikkiensis/pseudoavium/devriesei	0,1%
Gemmobacter sp. nov. (aquatilis)	0,1%
gen. nov. (Nocardioides solisilvae)	0,1%
gen. nov. (Sphingobacterium paludis)	0,1%
Janibacter alkaliphilus	0,1%
Kocuria carniphila	0,1%
Kocuria kristinae	0,1%
Lactobacillus dextrinicus	0,1%
Lactobacillus gasseri	0,1%
Lactobacillus harbinensis	0,1%
Lactobacillus pentosus/plantarum	0,1%
Lactococcus lactis ssp lactis	0,1%
Lysinibacillus sp. nov. (halotolerans)	0,1%
Macrococcus sp. nov. (caseolyticus)	0,1%
Microbacterium sp. nov. (flavum)	0,1%
Niabella sp. nov. (terrae)	0,1%
Niabella sp. nov. (yanshanensis)	0,1%
Paenibacillus barengoltzii	0,1%
Pseudoxanthomonas mexicana	0,1%
Rhodococcus qingshengii/jialingae	0,1%
Rummeliibacillus suwonensis	0,1%
Salana multivorans	0,1%
Sphingobacterium caeni	0,1%
Sphingobacterium hotanense	0,1%
Sphingobacterium nemaocida	0,1%
Staphylococcus arlettae	0,1%
Staphylococcus xylosus	0,1%
Streptococcus pluranimalium	0,1%
Tetragenococcus sp. nov. (solitarius)	0,1%
Tsukamurella carboxydivorans/ spumae/sunchonensis/pseudopumae	0,1%
Zimmermannella sp. nov. (alba)	0,1%
not identified	0,4%

Supplementary Table S5. Relative abundance (%) of families in samples 3 (farm A) and 4 (farm B) as detected by the cultivation-dependent (isolates) and -independent (amplicons) approach. Only families with a relative abundance of >1 % in at least one analysis or with detection in only the cultivation-dependent approach are listed.

	**Sample 3**		**Sample 4**	
**Family**	**Amplicon**	**Isolates**	**Amplicon**	**Isolates**
Acetobacteraceae			0.09	1.09
Aerococcaceae	3.79	2.98	9.49	6.90
Bacillaceae	1.23		1.68	1.36
Bifidobacteriaceae	0.18		2.66	0.27
Brevibacteriaceae	0.71	2.44	0.25	0.36
Burkholderiaceae	0.03		1.22	
Carnobacteriaceae	2.47	0.07	2.19	
Caulobacteraceae	0.03	0.21	0.43	1.27
Clostridiales Incertae Sedis XII	0.81		1.02	
Comamonadaceae	0.26	0.34	0.83	1.18
Corynebacteriaceae	9.88	8.54	9.96	12.89
Dermabacteraceae	1.21	3.12	1.73	2.00
Dietziaceae	0.83	1.56	0.14	
Enterococcaceae	0.12	0.75	0.42	1.18
Flavobacteriaceae	6.93	0.20	1.68	3.63
Gordoniaceae	0.18	6.51	0.10	0.18
Iamiaceae	1.23		0.02	
Intrasporangiaceae	6.27	3.39	0.25	0.09
Jonesiaceae		0.34		
Lachnospiraceae	7.05		4.23	
Lactobacillaceae	0.04	0.4	0.79	1.09
Leuconostocaceae	0.59	1.63	0.27	
Microbacteriaceae	1.14	11.59	0.93	31.67
Micrococcaceae	1.75	1.02	5.29	4.90
Moraxellaceae	1.37	0.21	1.31	0.54
Morganellaceae		0.07		
Mycobacteriaceae				0.36
Nocardioidaceae	0.34	0.07	0.05	1.45
Paenibacillaceae		0.07		0.27
Planococcaceae	0.27		14.97	0.18
Promicromonosporaceae	1.22	1.83	0.01	0.09
Propionibacteriaceae		0.95		0.54
Pseudomonadaceae	3.09	0.27	2.96	1.18
Rhizobiaceae	0.06		2.08	4.81
Rhodobacteraceae	1.05	0.07	0.44	0.09
Rhodocyclaceae	2.25		0.18	
Ruminococcaceae	6.91		3.59	
Saccharibacteria genera incertae sedis	1.13		0.98	
Sphaerobacteraceae	2.26		0.02	
Staphylococcaceae	11.34	37.97	5.43	2.72
Streptococcaceae	2.75	6.44	8.82	6.17
Tsukamurellaceae				0.09
Xanthomonadaceae	2.05	4.41	1.83	7.99

Supplementary Table S6. Genera and families (in bold) found in samples 3 and 4 by culturing but missed in the amplicon-based analysis. gen. nov., potential novel genus (the most similar genus is given in brackets). Genera exhibiting a mismatch with the 785R primer used in library PCR are marked with an asterisk (*).

**Phylum**	**Family**	**Genus**	**Relative abundance (%)**	
			Sample 3	Sample 4
Actinobacteria	Beutenbergiaceae	Salana		0.09
	Corynebacteriaceae	gen. nov. (Corynebacterium)		0.18
	Intrasporangiaceae	Arsenicicoccus	0.14	
	**Jonesiaceae**	Jonesia	0.34	
	Microbacteriaceae	Agromyces		0.64
		Curtobacterium	0.07	
		Plantibacter*	0.07	
		Zimmermannella		0.09
	Micrococcaceae	Citricoccus	0.34	
	**Mycobacteriaceae**	Mycobacterium		0.36
	Nocardioidaceae	gen. nov. (Nocardioides)		0.09
	Promicromonosporaceae	Cellulosimicrobium		0.09
	**Propionibacteriaceae**	Luteococcus*	0.75	
		Tessaracoccus*	0.07	0.54
		gen. nov. (Propioniciclava)*	0.14	0.09
	**Tsukamurellaceae**	Tsukamurella		0.09
Bacteroidetes	Sphingobacteriaceae	gen. nov. (Sphingobacterium)		0.09
Firmicutes	Aerococcaceae	gen. nov. (Aerococcus)	0.07	
	Enterococcaceae	Tetragenococcus		0.09
	**Paenibacillaceae**	Brevibacillus		0.18
		Paenibacillus	0.07	0.09
	Planococcaceae	Rummeliibacillus		0.09
Proteobacteria	Caulobacteraceae	Phenylobacterium	0.14	
	**Morganellaceae**	Proteus	0.07	
